# Use of drugs for ADHD among adults—a multinational study among 15.8 million adults in the Nordic countries

**DOI:** 10.1007/s00228-016-2125-y

**Published:** 2016-09-01

**Authors:** Øystein Karlstad, Helga Zoëga, Kari Furu, Shahram Bahmanyar, Jaana E Martikainen, Helle Kieler, Anton Pottegård

**Affiliations:** 1 Department of Pharmacoepidemiology, Norwegian Institute of Public Health, P.O.box 4404 Nydalen, 0403 Oslo, Norway; 2 Centre of Public Health Sciences, Faculty of Medicine, University of Iceland Reykjavik, Reykjavik, Iceland; 3 Centre for Pharmacoepidemiology, Department of Medicine, Karolinska Institute, Stockholm, Sweden; 4 Research Department, The Social Insurance Institution, Helsinki, Finland; 5 Clinical Pharmacology, Department of Public Health, University of Southern Denmark, Odense, Denmark

**Keywords:** ADHD, Psychostimulants, Adults, Pharmacoepidemiology, Nordic countries

## Abstract

**Purpose:**

The use of ADHD drugs among adults is controversial and has until recently not been approved for use in adults in most countries. The aim was to investigate use of ADHD drugs (stimulants and atomoxetine) among the entire adult population in the Nordic countries.

**Methods:**

We conducted a multinational population-based prescription register study based on the entire adult population in the five Nordic countries (Denmark, Finland, Iceland, Norway and Sweden). All users of ADHD drugs aged 18–64 years during 2008–2012 were included, which for 2012 comprised 76,896 drug users among 15.8 million adult inhabitants.

**Results:**

Annual prevalence of drug use increased during the study period for both genders and all age groups. The overall prevalence increased from 2.4 to 5.3 per 1000 men and 1.8 to 4.4 per 1000 women. Incidence also increased, but to a lesser extent in the last part of the study period. Methylphenidate was used by 88 % of drug users. Treatment was discontinued within the first year by 21 % of new drug users. Among all users of ADHD drugs, 53 % of men and 64 % of women concurrently used other psychotropic drugs, most frequently antidepressants and hypnotics. Psychotropic co-medication increased with age and was more pronounced among women than men.

**Conclusions:**

Use of ADHD drug among adults more than doubled over a 5-year period, and a majority were concurrently treated with other psychotropics. Adults constitute a substantial proportion of persons treated with ADHD drugs. Thus, evidence for long-term efficacy and safety in adults is urgently needed.

**Electronic supplementary material:**

The online version of this article (doi:10.1007/s00228-016-2125-y) contains supplementary material, which is available to authorized users.

## Introduction

The diagnosis of attention-deficit hyperactivity disorder (ADHD) among adults is controversial [1–4]. Nevertheless, evidence shows that symptoms of ADHD may persist into adulthood in a substantial proportion of child and adolescent patients [5–11]. The prevalence estimates of ADHD in adults are reported to be between 1 and 8 % [9], with variation by geographic region, diagnostic system used and underlying study population [6, 9, 12–14]. Adults with ADHD have a high degree of psychiatric comorbidity, complicating diagnostics as well as treatment [6, 15]. While physicians have previously had little guidance for diagnosing and treating adult ADHD, adults have been included in recent guidelines [8, 16–19], reflecting the increasing awareness of ADHD symptoms beyond childhood. However, until recently, atomoxetine was the only drug approved for use in adults [8, 9], which reflects that long-term efficacy and safety of these drugs are insufficiently studied in adults [20–22]. Despite these considerations, use of ADHD drugs among adults has increased rapidly throughout the world [14, 23–26]. To ensure rational use of stimulants and other ADHD drugs in the adult population, detailed knowledge on utilization patterns is thus urgently needed. Leveraging high-quality prescription register data available in the five Nordic countries, we aimed to describe the use of stimulants and atomoxetine in the adult population.

## Method

### Study setting, population and data sources

This population-based study examines the use of ADHD drugs among adults aged 18–64 years in ambulatory care in all five Nordic countries (Denmark, Finland, Iceland, Norway, Sweden), comprising in total 15.8 million inhabitants in this age range. Data on prescription drugs dispensed from pharmacies were retrieved from the nationwide prescription registers in each country [27]. Data on filled prescriptions are sent elec-tronically from all pharmacies to the national registers. Reporting to the registers is mandatory, ensuring high cover-age of prescription drug use in the ambulatory care setting. These registers allow individual-level drug use to be tracked over time by using the unique personal identity number (encrypted) assigned to all citizens [27].

The number of inhabitants in each country by gender and age group was retrieved from national population registers (Social Insurance Institution (Kela) in Finland and the bureau of statistics in the other countries). In 2012, the number of inhabitants aged 18–64 years was as follows: 3.4 million in Denmark, 3.3 million in Finland, 199,000 in Iceland, 3.1 million in Norway and 5.8 million in Sweden (total 15.8 million).

### ADHD drugs

Medical products in the Nordic countries are classified according to the Anatomical Therapeutic Chemical (ATC) classification system [28]. For the present study, ADHD drugs were defined as methylphenidate (ATC code N06BA04), atomoxetine (N06BA09), amphetamine (N06BA01) and dexamphetamine (N06BA02). Methylphenidate was further classified in extended release (ER) and immediate release (IR) formulations. Danish data did not include amphetamine, which is not marketed and thus rarely used in Denmark.

### Data analysis

Prescription data from each country were uploaded to a server at Statistics Denmark and analysed by a common programme in Stata version 13. Drug utilization was measured during 2008–2012, whereas data from 2006 to 2007 were used in specific analyses as a run-in period to define new users of ADHD drugs. Results are presented by gender and age attained at the start of the year by four age groups (18–24, 25–34, 35–44 and 45–64 years), combined for all Nordic countries (main results) and by country (Online Resource).

ADHD drug use was examined using the following definitions.

- *Prevalence*: Annual prevalence proportion (per 1000) of ADHD drug use was defined as the number of individuals who filled at least one prescription in one calendar year divided by the gender- and age-specific population of the same year.
- *Incidence*: Annual incidence (per 1000) of ADHD drug use was defined as the number of individuals who filled at least one prescription in one calendar year, and with no prescription fills during the previous 2 years (730 days), divided by the gender- and age-specific population of the same year.
- *Type of ADHD drug used at treatment initiation*: The type of ADHD drugs received at treatment initiation (i.e. first prescription fill) was assessed among new users during 2008–2012. New users were defined as individuals that had not previously filled any prescription for ADHD drugs (going back to 2006).
- *Early discontinuation and switch in drug treatment*: Among new users of ADHD drugs (defined as above), early discontinuation of treatment was defined as filling no more than two prescriptions for ADHD drugs during the first year (365 days) after initiating treatment. Prescriptions filled on the same date were counted as on prescription. Early switch in treatment was assumed if an individual who initiated treatment with methylphenidate filled a subsequent prescription for atomoxetine during the first year, or vice versa. In this analysis, users initiating treatment in 2012 were disregarded to ensure 1 year of prescription data follow-up.
- *Co-medication with other psychotropic drugs*: Use of other types of psychotropic drugs was examined among prevalent users of ADHD drugs. For all individuals who filled a prescription for an ADHD drug in one calendar year, the proportion (%) that filled prescriptions for other psychotropic drugs concurrently [29] with an ADHD drug (within 3 months) was calculated. Other psychotropic drugs were classified as antipsychotics (ATC N05A), anxiolytics (N05B), hypnotics and sedatives (N05C), antidepressants (N06A) and antiepileptics (N03A).

### Ethical approval

Personal identity numbers were encrypted. In accordance with laws and regulations, no ethical approval is needed for use of the Danish, Finnish and Norwegian prescription register data. The study was approved by the Icelandic Bioethics Committee (VSNb2013010018/03.07) and the Icelandic Data Protection Authority (2013010062TS/--), and in Sweden, it was approved by the the Karolinska Institutet regional ethics committee.

## Results

The source population was the Nordic population aged 18–64 years. In 2012, this comprised 15,828,232 inhabitants, with 5.3 per 1000 men (*n* = 42,450), 4.4 per 1000 women (*n* = 34,446) and 4.9 per 1000 in total (*n* = 76,896) filling a prescription for ADHD drugs.

### Prevalence

**Fig. 1:**
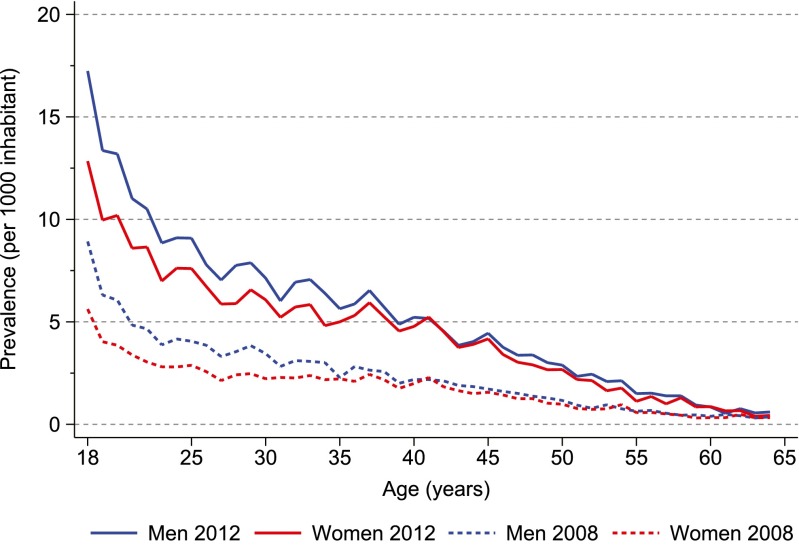
Annual prevalence proportion of ADHD drug use in 2008 and 2012 among persons aged 18–64 years in the Nordic countries, by 1-year age groups

The highest prevalence of ADHD drug use in 2012 was among 18-year olds at 17.2 per 1000 men and 12.8 per 1000 women (Fig. 1). Prevalence decreased with age in both genders. Substantial differences in prevalence were observed between countries (Online Resource Fig. S 1), with the highest prevalence in Iceland and the lowest in Finland (e.g. 25.2 versus 5.3 per 1000 among 18-year-old men).

**Fig. 2:**
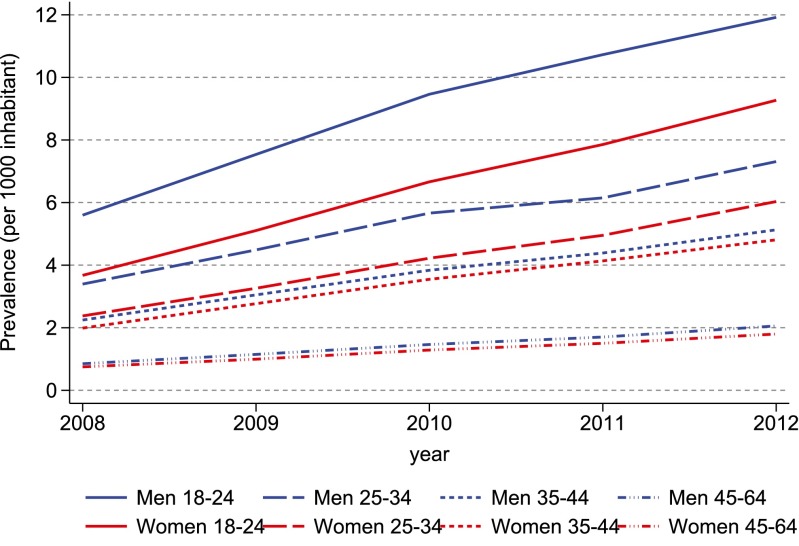
Annual prevalence proportion of ADHD drug use during 2008–2012 among persons aged 18–64 years in the Nordic countries, by year

From 2008 to 2012, the prevalence of ADHD drug use more than doubled in all gender and age categories (Fig. 2 and Online Resource Table S1), from 2.4 to 5.3 per 1000 men and from 1.8 to 4.4 per 1000 women aged 18–64 years. Thus, over the study period, the male/female ratio decreased from 1.33 to 1.20. Country-specific analyses showed increasing prevalence for all gender and age categories in all countries except Finland (Online Resource Fig. S2 and Table S1). The prevalence more than doubled in Iceland, Denmark and Sweden, while the increase in Norway was more modest.

Methylphenidate was the predominant drug dispensed, as it was used by 87–88 % of ADHD drug users in all age groups and both genders throughout the study period. A shift was observed from use of immediate release (IR) to extended release (ER) methylphenidate formulations; 64 vs 73 % of users received ER formulations in 2008 and 2012, respectively, while IR formulations decreased from 41 to 34 % in the same period. The proportion using atomoxetine remained stable at 12–14 %, while less than 5 % used amphetamine and dexamphetamine.

### Incidence

**Fig. 3:**
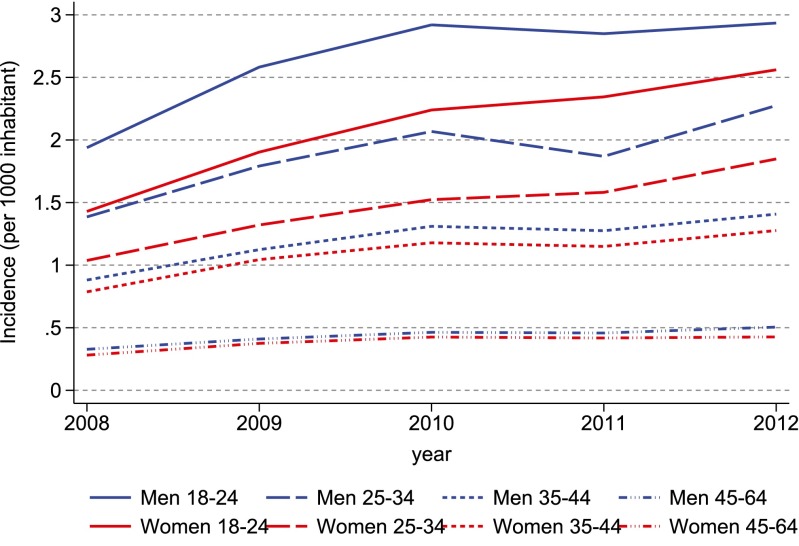
Annual incidence of ADHD drug use during 2008–2012 among persons aged 18–64 years in the Nordic countries, by year

The annual incidence of ADHD drug use increased modestly among all age groups and both genders during the study period, and mainly in 2008–2010 (Fig. 3). The changes in incidence over time varied by country, rising the most in Iceland and to a lesser extent in Denmark and Sweden, while remaining stable in Finland and Norway (Online Resource Fig. S3).

### Type of ADHD drug used at treatment initiation

**Fig. 4:**
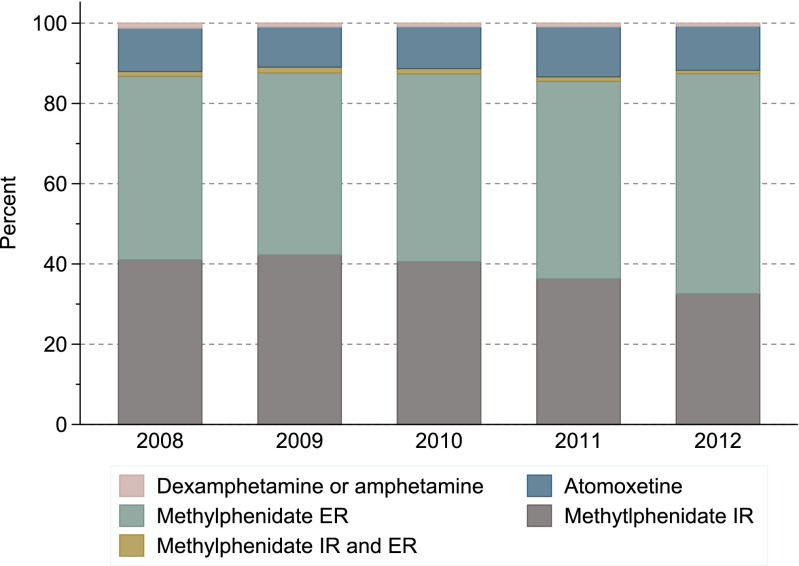
Type of ADHD drug used at treatment initiation during 2008–2012 among persons aged 18–64 years in the Nordic countries, by year. *IR* immediate release formulation, *ER* extended release formulation

Methylphenidate was the preferred drug at treatment initiation throughout the study period; 88 % of new users during 2008–2012 received methylphenidate on the first prescription fill, 11 % received atomoxetine, whereas amphetamine and dexamphetamine were rarely used (Fig. 4). In total, 38 % of the new users received IR methylphenidate formulations and 49 % received ER formulations. There was a shift towards using ER formulations as first-line treatment during the study period. These results were similar for the four age groups (data not shown). Substantial differences were observed between countries, as about 70 % initiated treatment on IR formulations in Denmark and Norway, whereas ER formulations were predominantly used in the other countries (Online Resource Fig. S4).

### Early discontinuation and switch in treatment

Among 62,144 new users of ADHD drugs during 2008–2011, 13 % filled only the initial prescription and 9 % filled only one more prescription, while 79 % filled 3 or more prescriptions of any ADHD drug during the first year of treatment. When restricting to users who initiated treatment on methylphenidate, 21 % filled only one or two prescriptions of any ADHD drug during the first year (discontinuation), while 8 % received atomoxetine within the first year (switching). Among users who initiated treatment on atomoxetine, 25 % filled only one or two prescriptions while 32 % received methylphenidate within the first year.

### Co-medication with other psychotropic drugs

Co-medication with other psychotropic drugs was common among ADHD drug users in all age groups and both genders (Table 1). Depending on gender and age category, 38–77 % of ADHD drug users received at least one other psychotropic drug concurrently with their ADHD medication. Co-medication was more common among older ADHD drug users and higher among women than men. Antidepressants were the most frequently used co-medication in all gender and age categories, highest among 45–64-year-old women (53 %). The proportion with co-medication was stable over the study period (data not shown).

**Table 1:** Proportion (%) of ADHD drug users with co-medication of other psychotropic drugs in 2012 among persons aged 18–64 years in the Nordic countries

Gender	Men					Women				
Age group	18–24	25–34	35–44	45–64	18–64	18–24	25–34	35–44	45–64	18–64
ADHD users (*n*)	14,396	11,972	9059	7023	42,450	10,693	9478	8210	6065	34,446
Any psychotropic	38	56	62	67	53	50	65	71	77	64
Antidepressants	20	32	37	40	30	32	44	49	53	43
Antiepileptics	7	15	16	17	13	10	17	19	21	16
Antipsychotics	14	20	20	19	18	13	18	18	19	17
Anxiolytics	7	17	21	25	16	13	23	26	32	22
Hypnotics	15	23	27	31	23	22	29	33	40	30
Melatonin	7	6	5	5	6	8	7	8	7	8
Other hypnotics	10	20	24	29	19	15	25	29	36	25

Proportion (%) of ADHD drug users with co-medication, defined as filling a prescription for other psychotropic drugs within 3 months before or after filling a prescription for an ADHD drug (i.e. concomitant use)

## Discussion

This multinational study provides a detailed overview of ADHD drug utilization among the entire adult population of the five Nordic countries. Use of ADHD drugs more than doubled during the 5-year study period, with 5.3 per 1000 men and 4.4 per 1000 women filling a prescription in the last year of the study. One in five patients discontinued ADHD drug use within the first year of treatment initiation. Co-medication with other psychotropics among users of ADHD drugs was common.

### Strengths and limitations

This study utilises data from high-quality prescription registers with mandatory reporting that cover the entire population of the Nordic countries [27]. Our results may slightly underestimate ADHD drug use, as the prescription registers do not include information on drugs administered in hospitals or institutions. The Finnish prescription register only covers reimbursed prescriptions and includes most prescriptions for ADHD drugs. However, during March 2011–March 2012, methylphenidate was not reimbursed for patients over 30 years. This may explain the slight dip in prevalence and incidence observed for Finland (Online Resource Figs. S2 and S3) but has little impact on the Nordic figures. Further, coverage for hypnotics is incomplete because melatonin is not reimbursable in Finland (Online Resource Table S2). Differences in licencing and reimbursement of psychotropic drugs and melatonin may impact the comparison of co-medication between countries. A limitation is the lack of information on the underlying ADHD diagnosis, which precludes solid conclusions on appropriateness of treatment.

### Licencing of ADHD drugs and types of drugs used

Licencing of ADHD drugs for use also in adults may partly explain the substantial increase in use observed in the present study. Methylphenidate has been extensively used in the treatment of ADHD in children and adolescents [14, 23, 25, 26, 30–32]. Over time, the labelling status for prescribing these drugs to both children and adults has varied between the Nordic countries. Continuation of atomoxetine treatment from childhood into adulthood was approved in Nordic countries in 2005, while continuation of methylphenidate treatment was approved from 2011 in some Nordic countries. After the study period of the present study (2008–2012), initiation of drug therapy in treatment-naïve adults has been approved for atomoxetine, methylphenidate and the new substance lisdexamphetamine.

Guidelines that also focus on the management of adult ADHD have only recently been published in all Nordic countries but Finland, stating that methylphenidate is first-line treatment while atomoxetine is second-line treatment [16–19]. Our study finds that methylphenidate was the preferred drug in both prevalent and new users as 9 of 10 patients used it. Use of atomoxetine remained limited, and dexamphetamine and amphetamine were rarely used. This differs markedly from the USA, where amphetamine and dexamphetamine formulations are used by a substantial proportion of adult patients [23, 31, 32]. Our study further revealed that the use of ER formulations of methylphenidate increased while IR formulations decreased. The ER formulation has the advantage of once-daily administration, which may be advantageous given the clinical manifestation of ADHD.

### Prevalence and incidence of ADHD drug use

The present study reveals a markedly increased prevalence of ADHD drug use during 2008–2012. However, the prevalences reported in the present study (2.1 per 1000 in 2008, 4.9 per 1000 in 2012) are substantially lower than what has been observed in the USA (12 per 1000 aged 20 years or older in 2005) [23], and higher than in the United Kingdom (1.1 per 1000 aged 18–24 years and 0.07 per 1000 aged 25–45 years in 2008) [24]. Differences in data source, the patient population under study and the definitions of drug use applied in each analysis may explain some of this geographical variation. Nevertheless, we found substantial difference in utilization patterns within the Nordic countries despite leveraging similar and nationwide data in five neighbouring countries, with similar healthcare systems and social structures, and used one programme for data analysis. Similarly, large variations have previously been observed between regions within the individual countries [33, 34]. These geographical variations in use point towards differences in guidelines [35], as well as prescribing practice and tradition being the most important parameters underlying the observed variations.

The prevalence of ADHD drug use increased substantially during the study period and was 4.9 per 1000 in 2012. Nevertheless, it is lower than the reported prevalence of the ADHD diagnosis, ranging from 10 to 80 per 1000 [6, 9, 12–14]. Our results also showed that the incidence of drug use increased, but to a lesser extent in the last part of the study period. Thus, the proportion of adults with ADHD not receiving pharmacological treatment may remain high. We found that drug use was more common in men than women, but the gender ratio was smaller than observed among children in the Nordic countries [30] and internationally [24, 26, 36]. The gender ratio for prescribing of ADHD drugs for adults in the UK is substantially higher than in the present study [24]. A diminishing gender difference from childhood to adulthood is in line with the reported differences by gender for diagnosis of ADHD [8].

### Early discontinuation and switch in treatment

Our results indicate that 21 % of persons who initiated treatment had discontinued all ADHD drugs within the first year. Other studies have revealed higher levels of discontinuation in adults [37–40]. Continuity of drug treatment may be compromised when patients transition from the adolescent health and social care systems to the adult system [10, 41], and some physicians may be reluctant to prescribe ADHD drugs to adults [1, 2, 4, 8]. We observed a higher proportion of early discontinuation and drug switching among users who initiated treatment with atomoxetine than those initiating treatment with methylphenidate. As atomoxetine is not considered first-line treatment [16–19], patients initiated directly on atomoxetine may differ from the overall study population, e.g. with respect to liability for substance misuse. Also, stricter regulation of substances classified as narcotics (methylphenidate) may lead to shorter supply per methylphenidate prescription, thus requiring more frequent prescription fills for methylphenidate than atomoxetine. Further studies should elucidate the long-term treatment duration and switch patterns for ADHD drugs.

### Co-medication of other psychotropic drugs with ADHD drugs

Studies of epidemiological and clinical populations of adults with ADHD have revealed that comorbid psychiatric illnesses are very common, in particular depression and substance use disorders [6, 8, 9, 15]. In the present study, antidepressants were the most frequently used psychotropic among users of ADHD drugs, with as many as half of the women over 34 years receiving antidepressants. Hypnotics were also commonly used, with the Z-hypnotics, such as zopiclone, preferred over melatonin. The presence of comorbidities poses additional challenges for diagnosis and treatment management of ADHD and may affect both the initiation and continuation of ADHD treatment [6, 15]. Evidence for the efficacy and safety of combined pharmacological treatment of ADHD and other psychiatric illnesses is limited.

### Unanswered questions

The present study did not reveal to what extent persons receiving pharmacological treatment with ADHD drugs also had a diagnosis for ADHD or other psychiatric illnesses, or vice versa. There is generally less evidence on the efficacy and safety for ADHD drugs in adults than in children, which is of concern given the substantially higher rates of comorbid psychiatric and somatic disorders in the adult population. For instance, ADHD drugs have been linked to a moderate increase in heart rate and blood pressure although evidence does not point to increased rate of serious cardiovascular events [42–45]. The Nordic countries provide an opportune setting for studies on these and other unanswered questions regarding outcomes of ADHD drug treatment in adults, with several health registers covering the entire population in each country and the possibility to track the exposures and outcomes of all persons over time [27].

### Conclusion

The present study revealed that the use of ADHD drug among adults more than doubled over a 5-year period, and a majority were concurrently treated with other psychotropics. Because of these recent developments in ADHD drug use patterns, adults now constitute about half of the persons using ADHD drugs in the Nordic countries. Thus, evidence for long-term treatment efficacy and safety in adults is urgently needed.

## Electronic supplementary material

ESM 1 (PDF 556 kb)
