# Supplementary material for: Use of drugs for ADHD among adults—a multinational study among 15.8 million adults in the Nordic countries
Source: Eur J Clin Pharmacol. 2016 Sep 1;72(12):1507–14. doi: 10.1007/s00228-016-2125-y (PMC5110707; doi:10.1007/s00228-016-2125-y)
Supplement: Supplementary file 1 — (PDF 556 kb) [file 228_2016_2125_MOESM1_ESM.pdf]

Online Resource

Supplementary material for article in European Journal of Clinical Pharmacology

Title: Use of drugs for ADHD among adults – a multinational study among 15.8 million adults in the Nordic countries

Authors: Øystein Karlstad, Helga Zoëga, Kari Furu, Shahram Bahmanyar, Jaana E Martikainen, Helle Kieler, Anton Pottegård

Correspondence: Øystein Karlstad, Department of Pharmacoepidemiology, Norwegian Institute of Public Health, P.O.box 4404 Nydalen, N-0403 Oslo, Norway. oystein.karlstad@fhi.no

Supplemental Figure S1: Annual prevalence proportion of ADHD drug use in 2012 per 1-year age group among persons aged 18-64 years, by Nordic country

a) Men

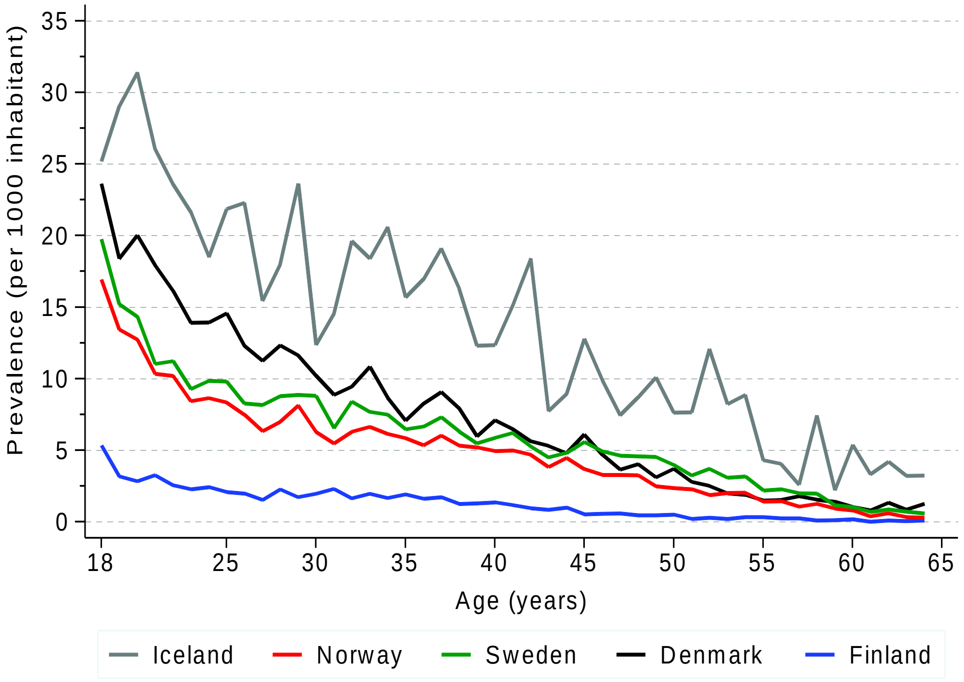

b) Women

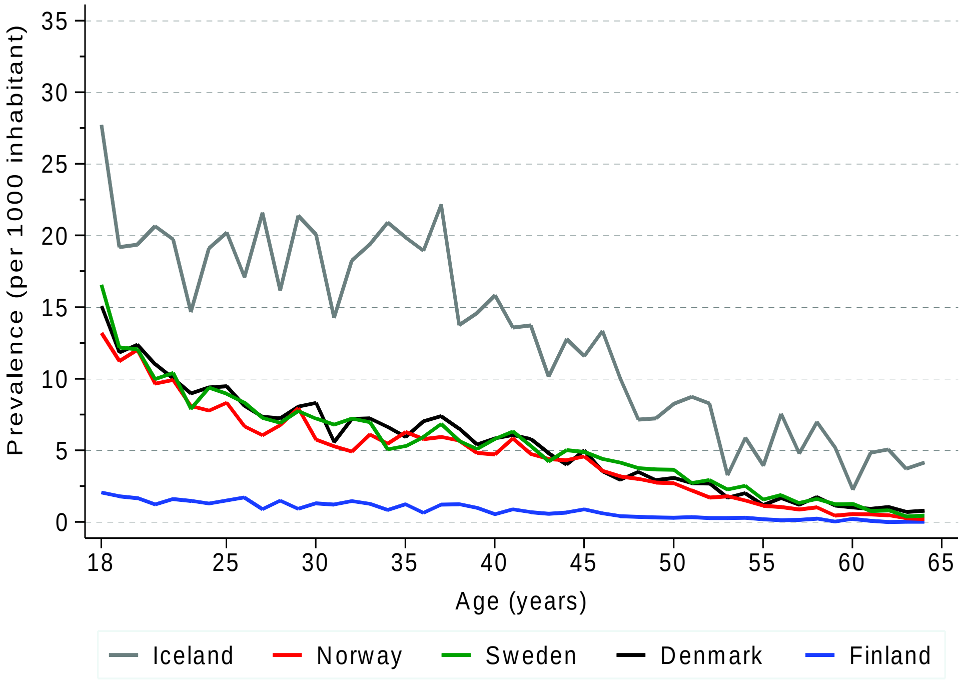

Supplemental Figure S2: Annual prevalence proportion of ADHD drug use during 2008-2012 among persons aged 18-64 years, by Nordic country

a) Men

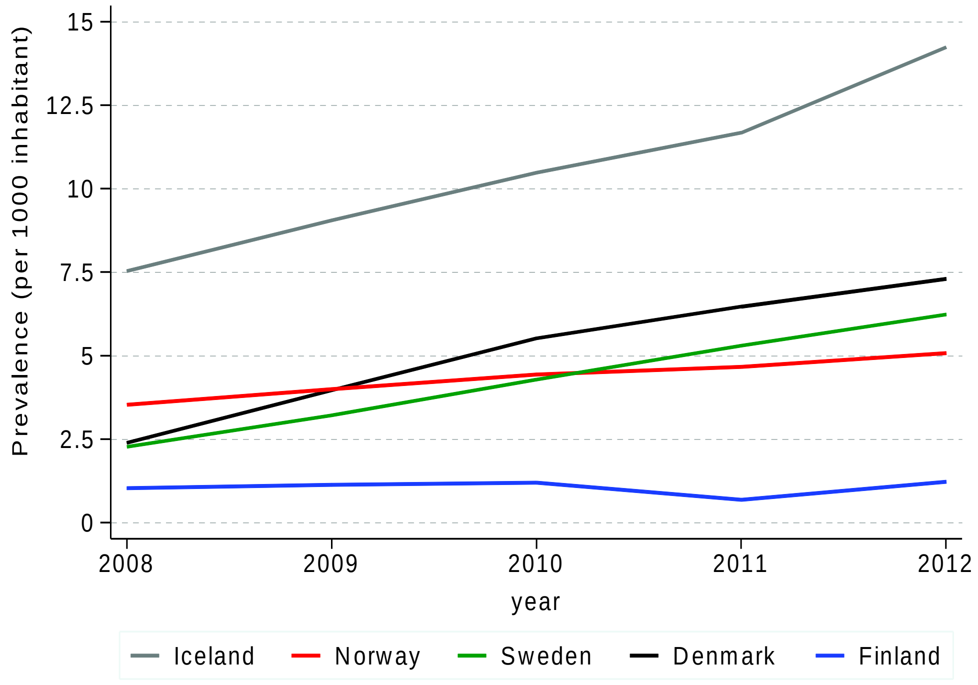

b) Women

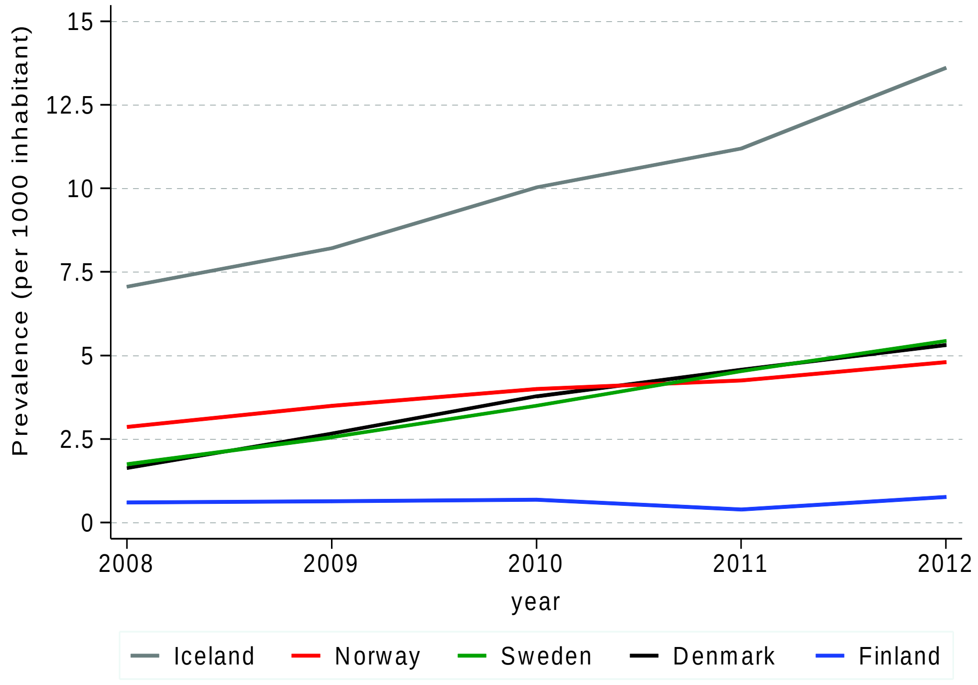

Supplemental Figure S3: Annual incidence of ADHD drug use during 2008-2012 among persons aged 18-64 years, by Nordic country

a) Men

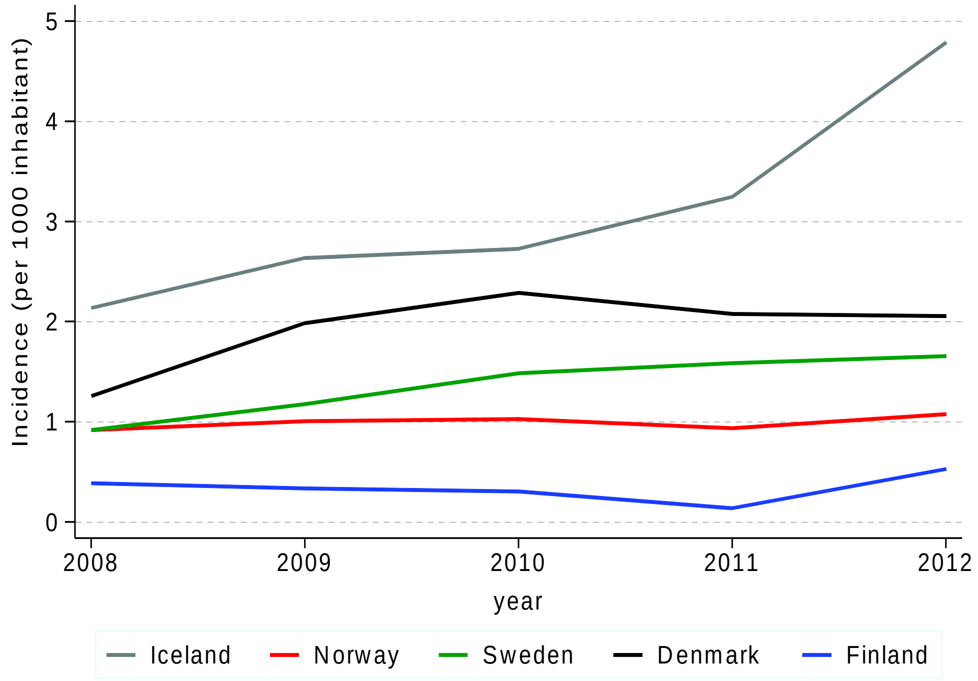

b) Women

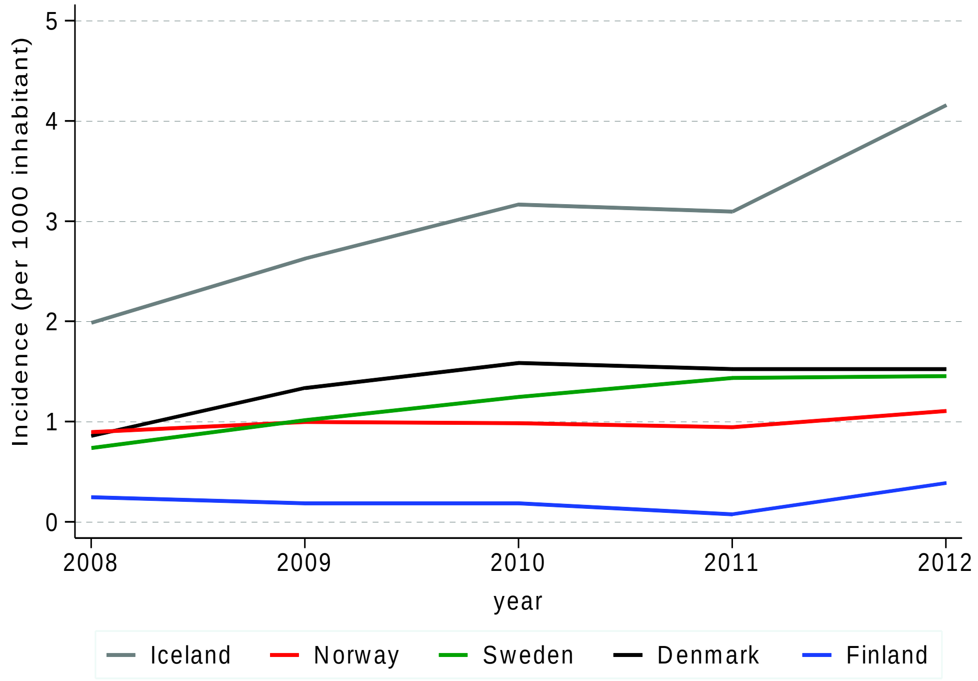

Supplemental Figure S4: Type of ADHD drugs used at treatment initiation during 2008-2012 among persons aged 18-64 years, by Nordic country

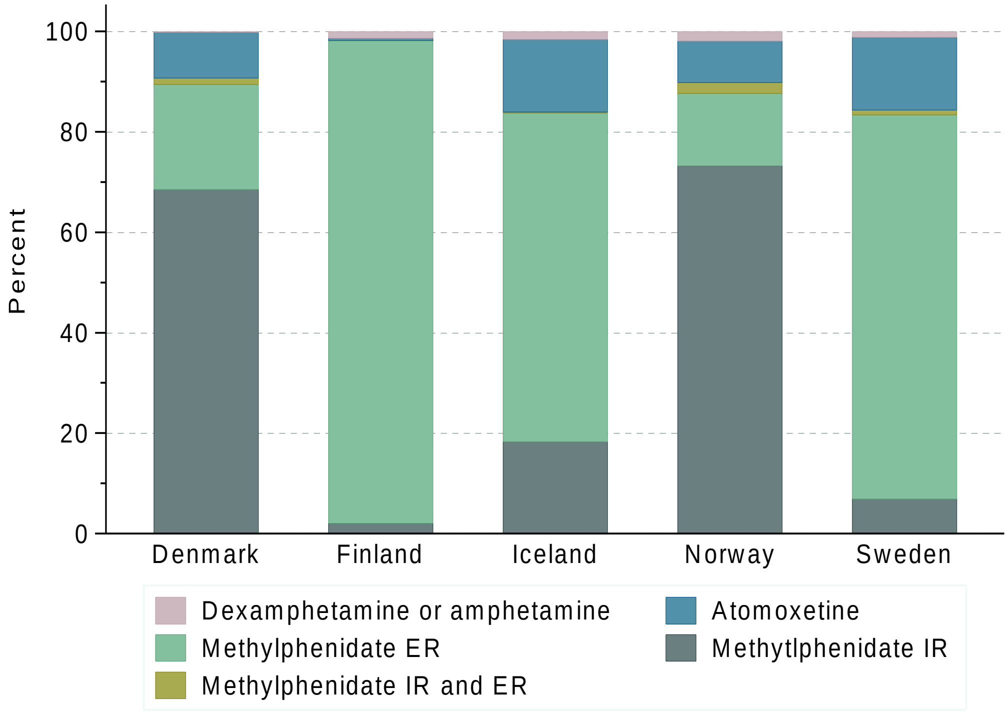

Abbreviations: IR: immediate release formulation; ER: extended release formulation.

Supplemental Table S1: Annual number of users (n) and prevalence proportion (n per 1000 inhabitant) of ADHD drug use among persons aged 18-64 years in 2008 and 2012, by Nordic country

Country and year	Men										Women									
	18-24		25-34		35-44		45-64		18-64		18-24		25-34		35-44		45-64		18-64	
	n	n/1000	n	n/1000	n	n/1000	n	n/1000	n	n/1000	n	n/1000	n	n/1000	n	n/1000	n	n/1000	n	n/1000
All																				
2008	6,113	5.6	5,458	3.4	4,087	2.2	2,848	0.9	18,506	2.4	3,833	3.7	3,690	2.4	3,481	2.0	2,479	0.7	13,483	1.8
2012	14,396	11.9	11,972	7.3	9,059	5.1	7,023	2.1	42,450	5.3	10,693	9.3	9,478	6.0	8,210	4.8	6,065	1.8	34,446	4.4
Denmark																				
2008	1,425	6.5	1,211	3.5	812	2.0	676	0.9	4,124	2.4	743	3.5	727	2.1	722	1.8	585	0.8	2,777	1.6
2012	4,453	17.8	3,591	11.0	2,627	6.7	1,875	2.5	12,546	7.3	2,712	11.3	2,433	7.5	2,266	5.9	1,623	2.2	9,034	5.3
Finland																				
2008	444	1.9	684	2.0	398	1.1	225	0.3	1,751	1.0	239	1.1	351	1.1	233	0.7	179	0.2	1,002	0.6
2012	749	3.1	677	1.9	445	1.3	221	0.3	2,092	1.2	369	1.6	426	1.3	284	0.9	211	0.3	1,290	0.8
Iceland																				
2008	234	14.2	199	7.9	174	7.5	173	4.5	780	7.5	146	9.3	188	8.4	171	8.0	170	4.7	675	7.1
2012	432	25.1	425	18.6	305	14.3	275	7.0	1,437	14.2	330	20.1	420	18.9	323	15.6	269	6.9	1,342	13.6
Norway																				
2008	1,911	9.2	1,555	5.0	1,188	3.3	650	1.1	5,304	3.5	1,331	6.7	1,157	3.8	1,099	3.2	567	1.0	4,154	2.9
2012	2,725	11.6	2,259	6.8	1,884	5.1	1,197	1.9	8,065	5.1	2,313	10.3	2,011	6.3	1,852	5.3	1,106	1.8	7,282	4.8
Sweden																				
2008	2,099	5.1	1,809	3.1	1,515	2.3	1,124	0.9	6,547	2.3	1,374	3.5	1,267	2.3	1,256	2.0	978	0.8	4,875	1.8
2012	6,037	13.0	5,020	8.3	3,798	5.9	3,455	2.8	18,310	6.2	4,969	11.2	4,188	7.3	3,485	5.6	2,856	2.4	15,498	5.4

Supplemental Table S2: Proportion (%) of ADHD drug users with co-medication of other psychotropic drugs in 2012 among persons aged 18-64 years, by Nordic country

	Denmark	Finland	Iceland	Norway	Sweden
**Men**					
Any psychotropic	50	53	51	43	60
Antidepressants	29	32	33	19	36
Antiepileptics	12	15	12	10	15
Antipsychotics	22	19	16	12	17
Anxiolytics	8	17	17	16	21
Hypnotics	13	15	18	18	32
Melatonin	7	0	1	7	6
Other hypnotics	8	15	17	14	29
**Women**					
Any psychotropic	62	59	64	51	72
Antidepressants	47	43	46	26	49
Antiepileptics	16	13	15	11	18
Antipsychotics	21	17	20	12	16
Anxiolytics	11	17	26	18	31
Hypnotics	19	15	24	24	41
Melatonin	10	0	2	10	7
Other hypnotics	11	15	23	17	38

Co-medication defined as filling a prescription for other psychotropic drugs within 3 months before or after filling a prescription for an ADHD drug, i.e. proportion (%) of ADHD drug users with co-medication.
